# Mendelian randomization provides evidence for a causal effect of higher serum IGF-1 concentration on risk of hip and knee osteoarthritis

**DOI:** 10.1093/rheumatology/keaa597

**Published:** 2020-10-07

**Authors:** April Hartley, Eleanor Sanderson, Lavinia Paternoster, Alexander Teumer, Robert C Kaplan, Jon H Tobias, Celia L Gregson

**Affiliations:** 1 MRC Integrative Epidemiology Unit, Population Health Sciences, Bristol Medical School, University of Bristol, Bristol, UK; 2 Musculoskeletal Research Unit, Translational Health Sciences, Bristol Medical School, University of Bristol, Bristol, UK; 3 Institute for Community Medicine, University Medicine Greifswald, Greifswald, Germany; 4 Department of Epidemiology and Public Health, Albert Einstein College of Medicine, New York, NY, USA

**Keywords:** OA, UK Biobank, insulin-like growth factor-1, Mendelian randomization, BMI

## Abstract

**Objectives:**

How insulin-like growth factor-1 (IGF-1) is related to OA is not well understood. We determined relationships between IGF-1 and hospital-diagnosed hand, hip and knee OA in UK Biobank, using Mendelian randomization (MR) to determine causality.

**Methods:**

Serum IGF-1 was assessed by chemiluminescent immunoassay. OA was determined using Hospital Episode Statistics. One-sample MR (1SMR) was performed using two-stage least-squares regression, with an unweighted IGF-1 genetic risk score as an instrument. Multivariable MR included BMI as an additional exposure (instrumented by BMI genetic risk score). MR analyses were adjusted for sex, genotyping chip and principal components. We then performed two-sample MR (2SMR) using summary statistics from Cohorts for Heart and Aging Research in Genetic Epidemiology (CHARGE) (IGF-1, *N* = 30 884) and the recent genome-wide association study meta-analysis (*N* = 455 221) of UK Biobank and Arthritis Research UK OA Genetics (arcOGEN).

**Results:**

A total of 332 092 adults in UK Biobank had complete data. Their mean (s.d.) age was 56.5 (8.0) years and 54% were female. IGF-1 was observationally related to a reduced odds of hand OA [odds ratio per doubling = 0.87 (95% CI 0.82, 0.93)], and an increased odds of hip OA [1.04 (1.01, 1.07)], but was unrelated to knee OA [0.99 (0.96, 1.01)]. Using 1SMR, we found strong evidence for an increased risk of hip [odds ratio per s.d. increase = 1.57 (1.21, 2.01)] and knee [1.30 (1.07, 1.58)] OA with increasing IGF-1 concentration. By contrast, we found no evidence for a causal effect of IGF-1 concentration on hand OA [0.98 (0.57, 1.70)]. Results were consistent when estimated using 2SMR and in multivariable MR analyses accounting for BMI.

**Conclusion:**

We have found evidence that increased serum IGF-1 is causally related to higher risk of hip and knee OA.

**Rheumatology key messages**

- Genetically determined serum insulin-like growth factor-1 is related to an increased risk of hip and knee OA.
- A high genetic risk for increased insulin-like growth factor-1 and BMI confers the highest risk for hip OA.
- Overall, results suggest a causal role of serum insulin-like growth factor-1 in hip and knee OA.

## Introduction

OA is highly prevalent, with an estimated 3.8% of the worldwide population affected by knee and 0.9% by hip OA [1]. Currently there are no disease-modifying drugs available; therapy consists of pain management and, when severe, joint replacement, with an estimated cost greater than £850 million in the UK for primary knee and hip replacement [2].

Insulin-like growth factor-1 (IGF-1) is a hormone regulating skeletal growth and development [3]. Most circulating IGF-1 is produced by the liver in response to growth hormone stimulation [3], whilst some is produced by specific tissues, e.g. chondrocytes [3, 4]. *In vitro* studies of animal cartilage suggest that IGF-1 can stimulate proteoglycan synthesis [5], upregulate type 2 collagen and downregulate MMP-13 expression [6], all of which imply that IGF-1 may be protective against cartilage degeneration (and hence OA). Epidemiological evidence supporting an IGF-1–OA association has been inconclusive [7], with the largest cross-sectional study (*N* = 761) identifying a positive association between IGF-1 concentration and bilateral knee OA [8]. A positive association between IGF-1 and OA risk is further supported by findings from individuals with acromegaly (a disorder of excess growth hormone production), who have increased OA risk [7]. Conversely, polymorphisms in the *IGF-1* promoter region, associated with lower IGF-1 levels, have been linked to higher OA prevalence [9, 10]. BMI is a strong risk factor for OA [11] and is inversely related to IGF-1 [12]; BMI may therefore mediate any inverse association between IGF-1 and OA.

Mendelian randomization (MR) enables causal inference in epidemiology. MR uses genetic variants, robustly associated with an exposure, as an instrument for the exposure, to determine the causal relationship with an outcome [13]. As genetic variants are randomly assigned at conception and cannot be changed, the genetic instrument(s) is generally independent of confounders of the exposure–outcome relationship and unaffected by reverse causality [13]. Frequently, the instrument(s) may be related to the outcome via a causal pathway not mediated by the exposure (i.e. horizontal pleiotropy), violating a key assumption of MR [13]. Hence, multivariable MR (MVMR) methods have been developed to estimate the direct causal effect of the exposure on the outcome when the instrument(s) may affect the outcome through another related exposure, provided the related exposure is included in the model, along with valid instruments for each exposure [14].

We aimed to utilize the large-scale availability of data for serum IGF-1 in the UK Biobank population to firstly determine the observational associations between IGF-1 and hospital-diagnosed OA at the hand, hip and knee, and then to use MR to determine the causal effect of circulating IGF-1 on OA at each joint. After this, we aimed to use MVMR to determine whether any observed causal effects are independent of BMI.

## Methods

### Observational analysis

#### UK Biobank population

UK Biobank is a UK-wide population of ∼500 000 people, aged 38–73 years, recruited during 2006–10 [15]. Participants provided a range of information (e.g. demographics, health status) via questionnaires and interviews; anthropometric measures and blood samples were collected (data available at: www.ukbiobank.ac.uk). A detailed description of the study design, participants and quality control (QC) methods is published elsewhere [15]. UK Biobank received ethical approval from the Research Ethics Committee (REC reference 11/NW/0382).

#### Measurement of serum IGF-1

Serum IGF-1 was measured at baseline using the Liaison XL chemiluminescent immunoassay, Diasorin Ltd (Dartford, UK) (data downloaded April 2019). Average within-laboratory coefficients of variation were 6.0% for low, 5.3% for medium and 6.2% for high concentrations [16]. QC procedures have been published [17].

#### Determination of hospital-diagnosed OA

Hand, hip and knee OA were determined from Hospital Episode Statistics [18] using the International Statistical Classification of Diseases and Related Health Problems (ICD) 9/10 codes previously reported for hand [19], hip and knee [20] OA (data downloaded January 2019). Inclusion (cases) and exclusion (controls) codes (to exclude controls with OA in other joints and inflammatory polyarthropathies) are listed in supplementary Table S1 A and B, available at *Rheumatology* online, respectively.

#### Covariates

BMI was determined from measured height and weight at the assessment clinic [weight (kg)/height (m)^2^]. Ethnic background (Supplementary Methods, available at *Rheumatology* online) and oestrogen replacement therapy (ERT) use were ascertained by touchscreen questionnaire.

#### Statistical analysis

Positively skewed serum IGF-1 concentrations were log-transformed, and associations with binary OA outcomes determined using multivariable logistic regression. Analyses were performed in four stages: (i) unadjusted; (ii) adjusting for age and sex; (iii) additionally adjusting for ethnicity and ERT use; and (iv) additional adjustment for BMI. Coefficients were transformed by ln(2) to generate an odds ratio (OR) per doubling in IGF-1 concentration. Results are presented as OR per s.d. increase in IGF-1 in figures to allow comparison with MR estimates. Additional stratified analyses determined gender-specific associations. We did not correct our *P-*value threshold for multiple testing as our three outcomes are highly correlated. We performed sensitivity analyses excluding individuals with acromegaly (ICD10 code E220, ICD9 code 2530), endocrine-related arthropathy (M145, 7130) (*N* = 94) and individuals for whom serum IGF-1 was measured from an aliquot other than the first aliquot (*N* = 43 728), as sample dilution issues have been reported by UK Biobank and the dilution increases with increasing aliquot [17].

### Causal inference using MR

#### Genotyping and imputation

Pre-imputation QC, phasing, imputation and QC filtering are described elsewhere [21, 22] and summarized in Supplementary Methods, available at *Rheumatology* online.

#### One-sample MR

**Fig. 1:**
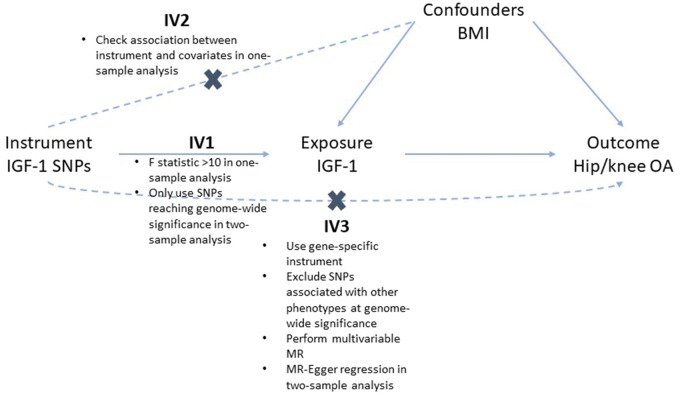
The assumptions of MR and how we tested these assumptions in our analyses For an MR effect estimate to be valid, the instrument(s) must satisfy three key assumptions [26]: IV1 [the instrument(s) must be robustly associated with the exposure]; IV2 [the instrument(s) must not be associated with any confounders of the exposure–outcome relationship]; and IV3 [the instruments(s) can only be associated with the outcome via the exposure and not via a different biological pathway independent of the exposure (i.e. horizontal pleiotropy)]. In one-sample analyses, IV1 was tested by calculating the F-statistic, which is a measure of instrument strength. A cut-off of ≥10 is used to determine sufficient instrument strength [13]. IV2 was tested by determining the association between the instruments and potential confounders of the exposure–outcome relationship. The Sargan statistic was used to detect evidence of potential pleiotropy; this statistic is a measure of variation in the outcome the instrument explains, independent of the exposure variable [14]. To limit potential horizontal pleiotropy, we repeated analyses excluding the SNPs also associated with IGF-BP3 at genome-wide significance (rs700753, rs646776, rs1065656) and using just the intronic IGF-1 SNP (rs978458) as the instrument. In 2S analyses, to satisfy IV1, we ensured that all instruments were robustly associated with the exposure by only including SNPs associated with the exposure at genome-wide significance. To address IV3, MR-Egger regression was performed to generate an estimate of horizontal pleiotropy. Cochran’s Q statistic was also calculated as a measure of potential pleiotropy. Weighted median regression was performed to determine the robustness of IVW estimates as weighted median estimates are valid even if up to 50% of the SNPs are not valid instruments [27]. To limit potential horizontal pleiotropy, we repeated analyses excluding the SNPs also associated with IGF-BP3. In MVMR, Sanderson-Windmeijer (S-W) conditional F-statistics were calculated for IGF-1 and BMI to determine conditional instrument strength [14]. MR: Mendelian randomization; SNP: single nucleotide polymorphism; IGF-1: insulin-like growth factor-1; IVW: inverse variance weighted.

Eight independent single nucleotide polymorphisms (SNPs) associated with IGF-1 at genome-wide significance, in the Cohorts for Heart and Aging Research in Genetic Epidemiology (CHARGE) meta-analysis, instrumented IGF-1 [23] (Table 1). Two of these SNPs were also associated with IGF-BP3 levels and one was identified from a bivariate analysis of IGF-1 and IGF-BP3. Analyses were performed using the individual SNPs (in the same analysis) and then an unweighted genetic risk score (GRS, generated by summing IGF-1-increasing allele dosage). One-sample (1SMR) analyses were adjusted for sex, genotyping chip and 10 principal components (PCs) (to account for population stratification, i.e. minor allele frequency variation due to ancestral differences). IGF-1 was standardized prior to analysis. Two-stage least-squares regression was performed using the ‘ivreg2’ package in Stata (StataCorp, College Station, Texas US) [24], which provides an estimate of the risk difference for a binary outcome. We generated an estimate of the OR per s.d. increase in IGF-1 by first regressing the instruments on IGF-1, generating predicted values of IGF-1, and then regressing these predicted values on the binary outcomes using logistic regression. The standard errors (SEs) for the OR estimate can be underestimated [25], but conclusions were the same when using the two-stage least-squares model (supplementary Table S2, available at *Rheumatology* online), therefore we present the OR estimates. A summary of the assumptions of MR, and how we tested these, is presented in Fig. 1. Power calculations for one-sample MR were performed using mRnd (http://cnsgenomics.com/shiny/mRnd/) [28] (supplementary Table S3, available at *Rheumatology* online).

**Table 1:** Associations of IGF-1 instruments for one-sample analyses with IGF-1 and OA in UK Biobank

SNP	EA	IGF-1			Hip OA			Knee OA		
		Beta	SE	*P*	OR	SE	*P*	OR	SE	*P*
rs1065656^a^	G	0.050	0.003	4 × 10^−84^	0.987	0.015	0.392	1.019	0.012	0.116
rs2153960	A	0.048	0.003	6 × 10^−76^	1.025	0.016	0.125	1.023	0.013	0.061
rs509035	A	0.054	0.003	5 × 10^−102^	0.998	0.015	0.897	1.011	0.012	0.342
rs646776^a^	T	−0.029	0.003	3 × 10^−25^	0.939	0.016	2 × 10^−4^	0.992	0.013	0.543
rs700753^a^	G	0.113	0.002	<1 × 10^−300^	0.982	0.015	0.234	0.984	0.011	0.155
rs780093	C	0.060	0.002	1 × 10^−135^	1.041	0.016	0.007	1.021	0.012	0.066
rs934073	G	0.035	0.003	4 × 10^−43^	1.011	0.016	0.471	1.000	0.012	0.988
rs978458	C	−0.074	0.003	6 × 10^−172^	0.953	0.015	0.003	0.983	0.012	0.180
IGF-1 GRS		0.058	0.001	<1 × 10^−300^	1.018	0.006	0.001	1.010	0.004	0.019

Adjusted for sex, genotyping chip and 10 principal components. Betas represent the per-effect allele increase in standardized IGF-1. ^a^SNPs also associated with IGF-BP3 in the CHARGE meta-analysis [23]. IGF-1: insulin-like growth factor-1; EA: effect allele; NEA: alternative allele; EAF: effect allele frequency; OR: odds ratio; SE: standard error; GRS: genetic risk score; CHARGE: Cohorts for Heart and Aging Research in Genetic Epidemiology.

#### Two-sample MR

SNP-exposure summary data were extracted from the CHARGE meta-analysis [23]. The study employed a sample-size weighted Z-score based meta-analysis due to assay heterogeneity across cohorts, hence betas and SEs could not be generated [23]. Betas were estimated from *P*-values [from the IGF-1 genome-wide association study (GWAS)] using the method of Rietveld *et al.* [29]*.* Summary statistics are shown in supplementary Table S4, available at *Rheumatology* online. To provide estimates of the SNP–outcome association, summary statistics for the IGF-1 SNPs were extracted from the largest GWAS meta-analysis of hip and knee OA to date, from UK Biobank and Arthritis Research UK OA Genetics (arcOGEN; a population with severe OA) [20]. Estimates for hand OA were generated by our own GWAS of hospital-diagnosed hand OA in UK Biobank, adjusting for sex, genotyping chip and 10 PCs, using a linear mixed model within the software ‘*BOLT*’ [30], as described in the published protocol [31]. Steiger filtering was performed to identify SNPs explaining a greater proportion of variance in OA sub-phenotypes compared with IGF-1 [32]. No SNPs were identified for exclusion. Summary statistics are presented in supplementary Table S4, available at *Rheumatology* online*.* Causal effects were estimated using inverse-variance weighted regression, performed using the TwoSampleMR R package [33]. MR-Egger regression was also performed to estimate possible bias due to directional pleiotropy, i.e. to provide valid causal estimates even if one of the key assumptions of MR was invalidated (Fig. 1). MR-Egger does not constrain the intercept of the regression line between the SNP–exposure and the SNP–outcome estimates at 0, and thus produces a valid estimate if the correlation between the direct SNP–outcome effect (i.e. the effect of a SNP on the outcome not mediated by the exposure) (IV3, Fig. 1) and the SNP-exposure effect (IV1, Fig. 1) is 0 [34]. The drawback of the method is the reduced statistical power. A summary of additional two-sample (2SMR) analyses, testing the assumptions of MR, is presented in Fig. 1.

#### Multivariable MR

We conducted one-sample MVMR to determine the BMI-independent causal effect of IGF-1 on OA. An unweighted BMI GRS was generated using the 63 independent SNPs (after linkage disequilibrium clumping with an *r*^2^ threshold of 0.001) from the Genetic Investigation of Anthropometric Traits (GIANT) consortium GWAS of the European sex-combined population [35] (supplementary Table S5, available at *Rheumatology* online, details the SNPs and their association with BMI in UK Biobank). Analyses were performed as for 1SMR, with the inclusion of BMI and the BMI risk score in the two-stage least-squares regression model. MVMR was also performed with height (instead of BMI) as a covariate (supplementary Tables S2 and Table S6, available at *Rheumatology* online).

#### Factorial MR

Factorial MR was used to determine whether there is an effect of high IGF-1 on OA risk, over and above the effect of high BMI. The MR population was stratified by the median for IGF-1 GRS and for BMI GRS and then categorized as those: (i) below the median for IGF-1 GRS and BMI GRS; (ii) above/equal to the median for IGF-1 GRS and below the median for BMI GRS; (iii) below the median for IGF-1 GRS and above the median for BMI; and (iv) above the median for both IGF-1 and BMI GRS. Logistic regression analysed GRS category (exposure) and OA variables (outcomes) adjusting for sex, genotyping chip and 10 PCs.

## Results

### Participant characteristics

In total, 421 527 individuals had complete data for observational analyses, of whom 332 059 (79%) had genetic data and were included in MR analyses (supplementary Fig. S1, available at *Rheumatology* online, details sample derivation). The mean (s.d.) ages of the observational and MR populations were 56.4 (8.1) and 56.5 (8.0) years, respectively. In both populations 54% were female, mean BMI was 27.3 (4.7) kg/m^2^ and IGF-1 concentration 21.5 (6) nmol/l (Table 2). In the observational population, 3.1% had hospital-diagnosed hip OA, 5.4% knee OA and 0.7% hand OA; respective proportions for the MR population were 3.2, 5.4 and 0.7%.

**Table 2:** Characteristics of the observational and MR study populations derived from the UK Biobank population

	Observational population (*N* = 421 527)		MR sub-population (*N* = 332 059)	
	Mean	s.d.	Mean	s.d.
Age, years	56.4	8.1	56.5	8.0
Height, cm	168.6	9.2	168.9	9.2
Weight, kg	77.9	15.8	78.0	15.8
BMI, kg/m^2^	27.3	4.7	27.3	4.7
IGF-1, nmol/l^a^	21.3	17.6, 24.9	21.3	17.6, 24.9
	*N*	%	*N*	%
Female	227 738	54.0	178 699	53.8
ERT use	84 341	37.0	67 181	37.6

Ethnicity				
White	401 844	95.3		
Black/Black British	6500	1.5		
Asian/Asian British	6489	1.5		
Chinese	1335	0.3		
Mixed	1619	0.4		
Other	3740	0.9		
Hospital-diagnosed OA				
Hip	12 425	3.1	9951	3.2
Knee	22 278	5.4	17 338	5.4
Hand	2727	0.7	2165	0.7

a Values represent median and interquartile range. ERT: oestrogen replacement therapy; MR: Mendelian randomization.

### Evidence from the observational data

In unadjusted analyses, increasing IGF-1 concentration was associated with lower odds of hand, hip and knee OA (Table 3), with the strongest association seen for hand OA [OR per doubling = 0.61 (95% CI 0.57, 0.65), *P* = 1.5 × 10^−58^]. Adjustment for age and sex reduced the strength of all associations, although evidence remained for a protective association of IGF-1 on all three OA outcomes. Further adjustment for ethnicity and ERT use did not alter observed associations. However, IGF-1 was strongly inversely associated with BMI in the UK Biobank population, with an s.d. increase in IGF-1 associated with a 0.13 s.d. decrease in BMI. When BMI was added to the model, only evidence suggesting a protective association of IGF-1 on hand OA [OR = 0.87 (0.82, 0.93), *P* = 4.2 × 10^−5^] remained. Whilst the association between IGF-1 and knee OA was fully attenuated by BMI adjustment, some evidence emerged for an increased odds of hip OA [OR = 1.04 (1.01, 1.07], *P* = 0.014]. There was evidence of an interaction between log-transformed IGF-1 and BMI [OR for interaction term = 1.02 (1.01, 1.03), *P* = 2 × 10^−6^].

**Table 3:** Observational associations between serum IGF-1 and hospital-diagnosed hip, knee and hand OA

	Hip OA (*N* = 398 965)			Knee OA (*N* = 408 872)			Hand OA (*N* = 389 308)		
Model	OR	95% CI	*P*	OR	95% CI	*P*	OR	95% CI	*P*
Unadjusted	0.71	0.69, 0.74	6.10 × 10^−108^	0.68	0.66, 0.69	2.47 × 10^−250^	0.61	0.57, 0.65	1.45 × 10^−58^
Adjusted for age and sex	0.94	0.91, 0.97	3.31 × 10^−4^	0.80	0.78, 0.82	6.98 × 10^−74^	0.80	0.75, 0.86	5.59 × 10^−11^
Adjust for age, ethnicity, ERT	0.94	0.91, 0.97	3.59 × 10^−4^	0.81	0.80, 0.83	1.63 × 10^−64^	0.82	0.77, 0.88	2.96 × 10^−9^
Adjusted for age, ethnicity, ERT, BMI	1.04	1.01, 1.07	0.014	0.99	0.96, 1.01	0.223	0.87	0.82, 0.93	4.21 × 10^−5^

ORs are per doubling in IGF-1 concentration. IGF-1: insulin-like growth factor-1; OR: odds ratio; ERT: oestrogen replacement therapy.

When BMI-adjusted analyses were stratified by sex, the association between IGF-1 and hip OA was only seen in females [OR_F_ = 1.07 (1.03, 1.12) *vs* OR_M_ = 1.00 (0.95, 1.05), supplementary Fig. S2, available at *Rheumatology* online]. The inverse association between IGF-1 and hand OA was seen with a similar magnitude in both sexes. Restricting analyses to 377 602 individuals whose IGF-1 was measured from their first aliquot did not alter conclusions drawn, nor did removing those with acromegaly, endocrine-related arthropathies or restricting to the MR population.

### Evidence from MR analyses

**Fig. 2:**
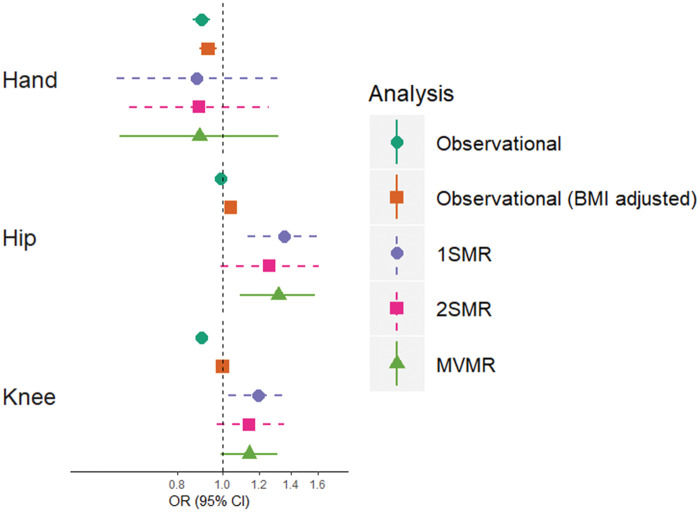
Comparison of observational and MR estimates of the effect of IGF-1 on hand, hip and knee OA Points represent odds ratios for OA per standard deviation increase in IGF-1 concentration. Horizontal bars represent 95% CIs. Observational analyses adjusted for age, sex, ERT, ethnicity and BMI. MR analyses adjusted for sex, genotyping chip and 10 principal components. OR: odds ratio; 1SMR: one-sample Mendelian randomization; 2SMR: two-sample Mendelian randomization; MVMR: multivariable Mendelian randomization; MR: Mendelian randomization; IGF-1: insulin-like growth factor-1; ERT: oestrogen replacement therapy.

In 1SMR analyses, using individual IGF-1-associated SNPs as instruments, we found evidence for an increased odds of hip OA with increasing IGF-1 concentration [OR per s.d. increase in IGF-1 = 1.20 (1.01, 1.43), *P* = 0.033]. Combining genotypes for the eight SNPs in a GRS strengthened the instrument (F-statistic: 3774 *vs* 563) and the evidence for a causal effect of IGF-1 on hip OA [OR = 1.35 (1.13, 1.63), *P* = 0.001, Fig. 2]. An effect of IGF-1 on knee OA was also observed when using the IGF-1 GRS instrument [OR = 1.19 (1.03, 1.37), *P* = 0.019]. Although we found no evidence of a causal effect of IGF-1 on hand OA [OR = 0.88 (0.60, 1.31), *P* = 0.539], these analyses were likely underpowered due to the rarity of hospital-diagnosed hand OA (supplementary Table S3, available at *Rheumatology* online). Evidence for a causal effect of IGF-1 on hip and knee OA was stronger when excluding the three SNPs also associated with IGF-BP3 levels [OR_hip_ = 1.57 (1.21, 2.02), *P* = 0.001 and OR_knee_ = 1.30 (1.07, 1.58), *P* = 0.008, supplementary Table S7, available at *Rheumatology* online]. The Sargan statistic was reduced from 30.5 (*P* < 0.001) to 4.4 (*P* = 0.35), suggesting that results were less biased by pleiotropy when excluding IGF-BP3 SNPs. Effects persisted when restricting to the single intronic *IGF-1* SNP [OR_hip_ = 1.93 (1.25, 2.97), *P* = 0.003 and OR_knee_ = 1.26 (0.90, 1.76), *P* = 0.179]. When stratifying by sex, stronger evidence for an effect of IGF-1 on hip OA was seen in females (supplementary Fig. S2, available at *Rheumatology* online), although analysis in males had lower power due to smaller sample size (supplementary Table S3, available at *Rheumatology* online). When checking the assumptions of 1SMR, we found evidence for an association between the IGF-1 GRS and both BMI and ERT use (supplementary Table S8, available at *Rheumatology* online), violating assumption IV2 (Fig. 1). Despite a strong inverse relationship between BMI and measured IGF-1, the association between the IGF-1 GRS and BMI was positive. We repeated 1SMR, adjusting for ERT use, which did not attenuate the association between IGF-1 and hip OA.

When determining the causal relationship using 2SMR, although CIs widened, effect sizes were similar (Fig. 2), with findings consistent with a positive effect of IGF-1 on hip OA [OR = 1.26 (0.99, 1.61), *P* = 0.065]. The MR-Egger estimate differed in direction of effect (supplementary Figs S3 and S4, available at *Rheumatology* online), suggesting horizontal pleiotropy may explain the observed association (Cochran’s Q = 19.6, *P* = 0.007). Further evidence for a potential pleiotropic effect was supported by two outlying SNPs (supplementary Fig. S4, available at *Rheumatology* online), rs646776 and rs700753; both were associated with IGF-BP3. When removing all three SNPs associated with IGF-BP3, the causal effect estimate for IGF-1 strengthened [OR = 1.49 (1.21, 1.83), *P* = 1 × 10^−4^] and was consistent in direction with the MR-Egger estimate [OR = 5.88 (0.70, 49.13), *P* = 0.200, *P* for intercept = 0.292, supplementary Table S7, available at *Rheumatology* online]. Cochran’s Q was also reduced (Q_hip_ = 4.4, *P* = 0.354 and Q_knee_ = 5.9, *P* = 0.206). We found no evidence of a causal effect of IGF-BP3 on hip or knee OA risk, but some evidence for a protective effect of IGF-BP3 on hand OA (supplementary Table S9, available at *Rheumatology* online). The effect of IGF-1 on hip OA was even stronger when restricting to the single intronic *IGF1* SNP [OR = 1.92 (1.22, 3.03), *P* = 0.005].

We postulated that BMI could mediate the effect of IGF-1 on hip OA; therefore, we performed MVMR to determine the causal effect of IGF-1 on hospital-diagnosed OA, independent of BMI. We found evidence for a BMI-independent causal pathway between IGF-1 and hip OA [OR = 1.32 (1.09, 1.58), *P* = 0.004], with weaker evidence for a causal effect on knee OA [OR = 1.14 (0.99, 1.31), *P* = 0.078, Fig. 2]. Evidence for a causal effect of IGF-1 on both hip and knee OA was stronger after excluding the IGF-BP3 SNPs (supplementary Table S7, available at *Rheumatology* online). The effect sizes for the causal role of IGF-1 on hip and knee OA were unchanged when performing MVMR with height instead of BMI (supplementary Table S2, available at *Rheumatology* online). Like univariable MR, a stronger effect of IGF-1 on hip OA was seen in females than males (supplementary Fig. S2, available at *Rheumatology* online), although this could be due to the smaller sample size of the male population.

**Fig. 3:**
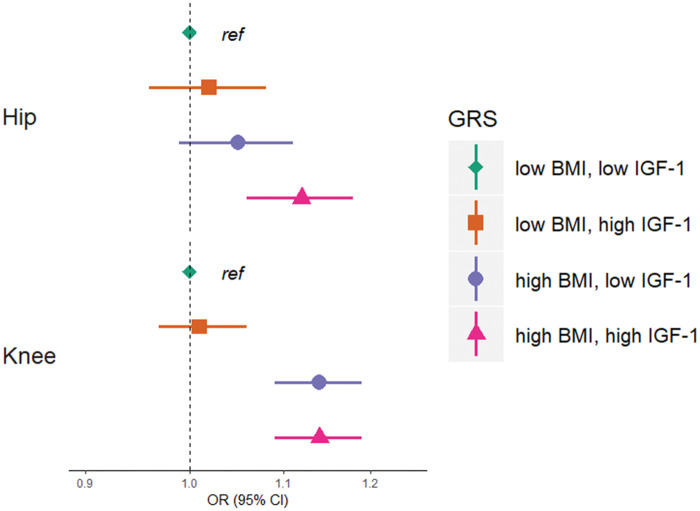
Factorial MR analysis of the interaction between IGF-1 and BMI on hip and knee OA risk Points represent the odds ratio for individuals in each BMI/IGF-1 GRS category compared with those with a BMI risk score below the median and an IGF-1 score below the median (reference category). Horizontal bars represent 95% CIs. Analyses adjusted for sex, genotyping chip and 10 principal components. MR: Mendelian randomization; IGF-1: insulin-like growth factor-1; GRS: genetic risk score.

We next performed factorial MR to identify any additive effect of IGF-1 and BMI on OA. Those with a BMI and IGF-1 GRS above the median had the greatest odds of hip OA [OR = 1.12 (1.06, 1.18), *P* = 1 × 10^−4^] compared with those with scores below the median (Fig. 3), suggesting an additive effect of higher serum IGF-1 and higher BMI on hip OA risk. No difference in the odds of knee OA was apparent between those with a high BMI GRS and low IGF-1 GRS *vs* those with a high IGF-1 GRS and high BMI GRS (Fig. 3). Results were similar when stratified by sex (supplementary Fig. S5, available at *Rheumatology* online).

In summary, our observational analyses provide evidence for a protective effect of higher serum IGF-1 on hand OA but an increased odds of hip OA after adjustment for BMI. An increased odds of hip OA is consistent with the MR analyses, which provided evidence for a causal effect of IGF-1 on hip and knee OA. Observational and factorial MR analyses both provided evidence for an interaction between high serum IGF-1 and high BMI on hip OA risk.

## Discussion

We have found evidence for a causal effect of higher circulating IGF-1 on the risk of hospital-diagnosed hip OA in a large population-based cohort of white European adults. This effect is independent of BMI. Both observational and MR analyses suggested that the effect of IGF-1 on hip OA is greater in those with a higher BMI, suggesting BMI modifies the effect of IGF-1 on hip OA. We detected evidence for a causal role of IGF-1 as a risk factor for knee OA, though this was weaker than that for hip OA. To the best of our knowledge, this is the first study to use MR to determine the causal relationships between IGF-1 and OA at the hand, hip or knee. Two prior studies have identified a positive relationship between a microsatellite polymorphism in the *IGF-1* promoter and radiographic hip OA [9, 10]. However, this polymorphism was also related to lower serum IGF-1 concentrations in a subset of 50 individuals [36], but the authors could not conclude that the SNP was the causal variant, or in linkage disequilibrium with a variant causing OA [10].

The lack of observational evidence for an association between IGF-1 and knee OA is consistent with a previous case–control study (Framingham Osteoarthritis Study) of both incident and progressive radiographic knee OA [37], and a cross-sectional analysis in the Baltimore Longitudinal Study of Aging [38]. Lloyd *et al.* [8] identified a positive association between IGF-1 and radiographic knee OA in the Chingford population, but only for severe and bilateral knee OA. Our phenotype of hospital-diagnosed OA is likely to reflect more severe radiographic or more clinically apparent (i.e. painful) OA. We lacked data on whether cases had bilateral or unilateral disease, which may contribute to the inconsistency in findings. Furthermore, Lloyd *et al.* [8] found weak evidence for increased serum IGF-1 in individuals with radiographic DIP joint OA, which contrasts with the protective association between IGF-1 and hand OA that we observed. Although the age-standardized prevalence of radiographic hand OA was 27% in the US Framingham population [39], UK hospital-diagnosis was much rarer, likely due to the lack of surgical management options for hand OA, meaning our MR analyses of hand OA were underpowered to confirm or refute the reported effect.

Consistent with a role of the IGF-1/IGF-BP axis on hip OA risk, a GWAS of hip OA identified two loci near *IGF-BP3* [40] where SNPs were associated with a decreased odds of hip OA and decreased circulating IGF-BP3 (not IGF-1) [40]. *In vivo* functional studies suggest that IGF-BP3 overexpression in cartilage from patients with knee OA results in decreased aggrecan and increased MMP-13 expression, two markers of cartilage degradation [40]. The two OA-associated SNPs near *IGF-BP3* were not instruments in our analyses, nor in linkage disequilibrium with any of the SNPs used in our instrument. Our two-sample MR analyses did not suggest a causal effect of circulating IGF-BP3 on hip OA risk.

The lack of consistency between our observational and MR results may reflect the difference in exposures; for observational analyses, the exposure was current measured IGF-1 levels whereas for MR analyses, the exposure was genetically predicted IGF-1 levels [41]. Different relationships of measured IGF-1 and the IGF-1 GRS with BMI may be explained by a negative feedback loop, whereby higher IGF-1 levels throughout the lifecourse lead to a higher body mass, which, over a sustained period of time, may reduce IGF-1 production by the liver [12]. However, the BMI GRS was not associated with current IGF-1 levels [β = 3.12 × 10^−4^ (95% CI –0.004, 0.004)]. We therefore hypothesize that higher IGF-1 throughout the life-course may drive the progression of OA, and our MVMR analyses suggest that this effect is not mediated by BMI. One potential pathway is via increased bone mineral density (BMD), a reported risk factor for hip OA [42]. IGF-1 contributes to skeletal development and increases BMD by promoting mesenchymal stem cell differentiation into osteoblasts [43]. However, adjustment for BMD did not attenuate our observational relationship between IGF-1 and hip OA (data not shown). An alternative explanation is that increased IGF-1 during development may lead to alterations in hip shape. IGF-1 is important for endochondral bone formation [44] and several genes linked to endochondral bone formation were identified in a recent GWAS meta-analysis of hip shape [45]. Variation in hip shape is associated with hip OA [46]. A cohort with IGF-1 and hip shape measured prior to OA onset (i.e. an adolescent cohort) is needed to better understand this relationship. As we observed a stronger effect of IGF-1 on hip OA in females, we further hypothesized that IGF-1 levels could lower circulating oestrogen levels, leading to increased OA risk, as oestrogen may be protective against OA [47]. However, the IGF-1 GRS was not related to menopausal age. Another potential explanation for the differences between the observational and MR results could be additional unmeasured confounding, biasing the observational analyses [41]. The potential for confounders to strongly bias observational results is highlighted by the difference in direction of effect observed for hip OA, before and after adjustment for BMI. As long as the instrument used for MR analysis meets the three key assumptions of MR (highlighted in Fig. 1), the MR estimate is not biased by confounding [13] and therefore we have more confidence in our estimates generated by MR.

A major strength of this analysis is the availability of extensive data for both IGF-1 concentrations and hospital diagnosed OA for >400 000 individuals, making this the most well-powered study to determine the observational relationship between IGF-1 and OA, to date. Furthermore, we had genotype data available for >300 000 individuals, providing 80% power to detect a causal OR of >1.28. The availability of these genetic data enabled us to perform one-sample MVMR analysis to determine the true causal effect of IGF-1 on hip OA, independent of BMI. However, we acknowledge limitations within these analyses. Although we excluded controls with other diagnosed arthropathies, some may still have had undiagnosed OA, although this would likely bias results towards the null. Our sex-stratified and hand OA analyses had low power, meaning we are unable to draw robust conclusions. The effect sizes of the summary statistics for the SNP–IGF-1 associations, used for two-sample MR analyses, were approximated as an s.d. unit change in IGF-1 from the corresponding *P*-values, direction of association, sample size and allele frequency [29]. However, these effect estimates were not used for 1SMR, which generated consistent results. We chose not to generate our BMI instrument from the largest GWAS of BMI, as a large proportion of individuals included in this meta-analysis were from UK Biobank and we were concerned about overestimating causal effect estimates due to ‘Winner’s-curse bias’ [48, 49]. We acknowledge that dichotomizing the population based on their GRS may not be the most efficient method for performing factorial MR and we cannot rule out a possible unobserved interaction between IGF-1 and BMI on knee OA risk. Recently, an alternative method was proposed for greater power in factorial MR analyses, using the complete set of instruments and their interactions [50]. The overall UK Biobank population is predominantly white British, with a higher prevalence of home-owners and non-smokers, a lower BMI and fewer self-reported health concerns than the general population [51], and MR analyses were restricted to those of white European ancestry, limiting generalizability. ‘Survivor bias’ may explain associations observed; if higher IGF-1 levels are related to a lower mortality risk, those with higher IGF-1 levels will be surviving long enough to develop chronic diseases such as OA. However, IGF-1 levels appear independent of all-cause mortality [52]. The UK Biobank population is limited by latent population structure even after restricting to white Europeans and adjusting for PCs [53], which may confound the relationship between IGF-1 and hospital-diagnosed hip OA. The discrepancy between observational and MR analyses most likely reflects unmeasured confounding, highlighting the utility of MR.

We identified robust evidence that higher concentrations of serum IGF-1 are a causal risk factor for hip OA in a very large UK population, with some evidence for a causal role in knee OA, and no evidence for an association with hand OA. Our MVMR analyses suggest that this causal role is independent of BMI, consistent with our observational analyses for hip, but not knee, OA. Further study is justified to determine the mechanism underlying this relationship.

## Supplementary Material

keaa597_Supplementary_Data
