# Supplementary material for: Mendelian randomization provides evidence for a causal effect of higher serum IGF-1 concentration on risk of hip and knee osteoarthritis
Source: Rheumatology (Oxford). 2020 Oct 7;60(4):1676–86. doi: 10.1093/rheumatology/keaa597 (PMC8023994; doi:10.1093/rheumatology/keaa597)
Supplement: keaa597_Supplementary_Data [file keaa597_supplementary_data.zip › keaa597-suppl_data/rhe-20-0365-File005.docx]

**SUPPLEMENTARY MATERIAL**

# Supplementary Methods

**Covariate data in UK Biobank**

Ethnicity was categorised as White, Mixed (White and Black Caribbean, White and Black African, White and Asian or any other Mixed background), Asian or Asian British, Black or Black British, Chinese or other ethnic group.

**Genotyping and imputation in UK Biobank**

The UK Biobank full data release contains data on all successfully genotyped samples (n=488,377). 49,979 individuals were genotyped using the UK BiLEVE array and 438,398 the UK Biobank axiom array. In brief, prior to phasing, multiallelic and rare SNPs (minor allele frequency [MAF]≤1%) were removed. Phasing of genotype data was performed using a modified version of the SHAPEIT2 algorithm (1). Genotype imputation to a reference set, combining the UK10K haplotype and HRC reference panels (2), was performed using IMPUTE2 algorithms (3). Analyses were restricted to autosomal variants by graded filtering with varying imputation quality according to allele frequency ranges, rarer genetic variants were required to have a higher imputation info score (info>0.3 for MAF >3%; info>0.6 for MAF 1-3%; info>0.8 for MAF 0.5-1%; info>0.9 for MAF 0.1-0.5%) with MAF and info scores having been recalculated on an in-house derived ‘European’ subset.

Individuals with mismatches between genetic and reported sex or individuals with sex chromosome aneuploidy were excluded. The sample was restricted to individuals of European ancestry as defined by an in-house k-means cluster analysis performed using the first 4 principal components (PCs), provided by UK Biobank, in R. The current analysis uses the largest cluster from this analysis. Estimated kinship coefficients, using the KING toolset (4), identified pairs of related individuals (1). An inhouse algorithm preferentially removed individuals related to the greatest number of other individuals, until no related pairs remained (16% removed).

## **Supplementary Table S1A: ICD codes used to identify cases of hospital-diagnosed hip, knee and hand OA**

| **Joint** | **Code** | | **Description** |
| --- | --- | --- | --- |
|  | **ICD10** | **ICD9** |  |
| Hand | M151 |  | “Herberden nodes (with arthropathy)” |
|  | M152 |  | “Bouchard nodes (with arthropathy)” |
|  | M154 |  | “Erosive (osteo)arthrosis” |
|  | M180 |  | “Primary arthrosis of first carpometacarpal joints, bilateral” |
|  | M181 |  | “Other primary arthrosis of first carpometacarpal joint” |
|  | M189 |  | “Arthrosis of the first carpometacarpal joint, unspecified” |
|  | M1904 |  | “Primary arthrosis of other joints (hand)” |
|  | M1994 |  | “Arthrosis, unspecified (hand)” |
|  |  | 71534 | “Osteoarthrosis, localized, primary or secondary, hand” |
|  |  | 71514 | “Osteoarthrosis, localized, primary, hand” |
| Hip | M16 |  | “Coxarthrosis” |
|  | M160 |  | “Primary coxarthrosis, bilateral” |
|  | M161 |  | “Other primary coxarthrosis” |
|  | M169 |  | “Coxarthrosis, unspecified” |
|  | M1905 |  | “Primary arthrosis of other joints (pelvic region and thigh)” |
|  | M1995 |  | “Arthrosis, unspecified (pelvis region and thigh)” |
|  |  | 71535 | “Osteoarthrosis, localized, primary or secondary, pelvic region and thigh” |
|  |  | 71515 | “Osteoarthrosis, localized, primary, pelvic region and thigh” |
| Knee | M17 |  | “Gonarthrosis” |
|  | M170 |  | “Primary gonarthrosis, bilateral” |
|  | M171 |  | “Other primary gonarthrosis” |
|  | M179 |  | “Gonarthrosis, unspecified” |
|  | M1906 |  | “Primary arthrosis of other joints (lower leg)” |
|  | M1996 |  | “Arthrosis, unspecified (lower leg)” |
|  |  | 71536 | “Osteoarthrosis, localized, primary or secondary, lower leg” |
|  |  | 71516 | “Osteoarthrosis, localized, primary, lower leg” |

## **Supplementary Table S1B: ICD codes used to exclude controls with OA at other sites or other arthropathies for hospital-diagnosed hip, knee and hand OA**

|  |  | **ICD10** | | | | | | | **ICD9** | | | | |  |
| --- | --- | --- | --- | --- | --- | --- | --- | --- | --- | --- | --- | --- | --- | --- |
| M111*  M112*  M118*  M119*  M13  M130  M1300  M1305  M1306  M1309  M131  M1310  M1314  M1315  M1316  M1319  M138  M1380  M1384  M1385  M1386  M1389  M139  M1390  M1394  M1395  M1396  M1399  M15  M150  M151 | M152  M153  M154  M158  M159  M1599  M16  M160  M161  M162  M163  M164  M165  M166  M167  M169  M17  M170  M171  M172  M173  M174  M175  M179  M18  M180  M181  M182  M183  M184  M185 | M189  M19  M190*  M191*  M192*  M198*  M199*  M20  M200  M210  M2100  M2105  M2106  M2109  M211  M2110  M2115  M2116  M2119  M212  M2120  M2124  M2125  M2126  M2129  M22  M220  M221  M222  M223  M224 | M228  M229  M23  M230  M2300  M2301  M2302  M2303  M2304  M2305  M2306  M2307  M2309  M231  M2310  M2311  M2312  M2314  M2315  M2316  M2317  M2319  M232  M2320  M2321  M2322  M2323  M2324  M2325  M2326  M2327 | M2329  M233  M2330  M2331  M2332  M2333  M2334  M2335  M2336  M2337  M2339  M234  M2340  M2341  M2342  M2343  M2344  M2345  M2346  M2347  M2349  M235  M2350  M2351  M2352  M2353  M2354  M2355  M2356  M2357  M2359 | M236  M2360  M2361  M2362  M2363  M2364  M2365  M2366  M2367  M2369  M238  M2380  M2381  M2382  M2383  M2384  M2385  M2386  M2387  M2389  M239  M2390  M2391  M2392  M2393  M2394  M2395  M2396  M2397  M2399  M24 | M240*  M241*  M242*  M243  M2430  M2434  M2435  M2436  M2439  M244  M2440  M2444  M2445  M2446  M2449  M245  M2450  M2454  M2455  M2456  M2459  M246  M2460  M2464  M2465  M2466  M2469  M247*  M248  M2480  M2484 | M2485  M2486  M2489  M249  M2490  M2494  M2495  M2496  M2499  M25  M255*  M256*  M257*  M258*  M259*  M42  M420*  M421*  M429*  M472*  M478*  M479  M4790  M4791  M4792  M4793  M4794  M4795  M4796  M4797  M4798 | M4952  M4953  M4954  M4955  M4956  M4957  M4958  M4959  M498*  M50  M501  M502  M503  M508  M509  M51  M949*  Q65*  V134  V135  V136  M21 | | 712  7121  7122  7123  7128*  7129*  715  7150  7151*  7152*  7153*  7158  7159  7161*  7165  71650  71654  71655  71656  71659  7166  71660  71664  71665  71666  71669  7168*  7169*  717  7170  7171 | 7172  7173  7174  7175  7176  7178  7179  718  7180*  7181*  7182*  7183*  7184*  7185*  7186*  7188*  7189*  7192*  7194*  7195*  7196*  7197  7198*  7199*  7200  721*  722  7220  7221  7222  7223 | 7224  7225  7226  7229  723  7230  7231  7295*  73100  73101  73102  73103  73104  732  7320  7321  7322  7323  7324  7325  7326  7327*  7328*  7329  7339*  736  7362  7363  7364  7365  7366 | 7367  7368  7369  7543  75430  75431  75432  7544  75440  75441  75442  75443  75444  7545  75450  75451  75452  75453  75459  7546  75460  75461  75469  835  8350  8351  836  8360  8361  8362  8366 | |
|  |  |  |  |  |  |  |  |  | |  |  |  |  | |

ICD: International Statistical Classification of Diseases and Related Health Problems

* represents a wildtype character representing every number from 1 to 9

## **Supplementary Table S2: one-sample univariable and multivariable MR results generated using two-stage least squares regression**

|  | **Univariable MR: IGF-1** | | | **Multivariable MR: IGF-1** | | | **Multivariable MR: BMI** | | |
| --- | --- | --- | --- | --- | --- | --- | --- | --- | --- |
|  | **risk difference** | **95% CI** | ***P*** | **risk difference** | **95% CI** | ***p*** | **risk difference** | **95% CI** | ***p*** |
| Hand OA | -0.001 | -0.004, 0.002 | 0.534 | -0.001 | -0.004, 0.002 | 0.554 | -0.001 | -0.003, 0.002 | 0.601 |
| Hip OA | 0.009 | 0.004, 0.015 | 0.001 | 0.009 | 0.003, 0.014 | 0.003 | 0.015 | 0.010, 0.021 | <0.001 |
| Knee OA | 0.009 | 0.001, 0.016 | 0.019 | 0.006 | -0.001, 0.014 | 0.081 | 0.035 | 0.029, 0.042 | <0.001 |
|  | **Univariable MR: IGF-1** | | | **Multivariable MR: IGF-1** | | | **Multivariable MR: Height** | | |
| Hand OA | -0.001 | -0.004, 0.002 | 0.534 | -0.001 | -0.004, 0.002 | 0.542 | -2.98x10^-5^ | -0.002, 0.001 | 0.968 |
| Hip OA | 0.009 | 0.004, 0.015 | 0.001 | 0.009 | 0.003, 0.015 | 0.002 | 0.001 | -0.002, 0.005 | 0.344 |
| Knee OA | 0.009 | 0.001, 0.016 | 0.019 | 0.008 | 0.001, 0.015 | 0.029 | 0.003 | -0.001, 0.007 | 0.106 |

Risk differences are per SD increase in IGF-1. F-statistics for 1SMR and S-W conditional F statistics for MVMR>3000.

MVMR with height was performed as for BMI, with height instrumented by 292 independent height SNPs (see *Supplementary Table S*7 for a list of SNPs used to instrument height) from the GIANT meta-analysis of 253,288 individuals (5)*.*

MR: Mendelian randomization; BMI: body mass index; CI: confidence intervals; OA: osteoarthritis

## **Supplementary Table S3: Power estimates for Mendelian randomisation analyses**

|  | | **Power** | | |
| --- | --- | --- | --- | --- |
|  | | **Total population**  **(N=332,092)** | **Female population**  **(N=178,719)** | **Male population**  **(N=153,373)** |
| **Hand OA (K=0.007)^a^** | |  |  |  |
|  | OR=0.95 | 0.06 | 0.06 | 0.05 |
|  | OR=0.90 | 0.08 | 0.07 | 0.06 |
|  | OR=0.80 | 0.16 | 0.13 | 0.08 |
|  | OR=0.70 | 0.30 | 0.24 | 0.12 |
|  | OR=0.60 | 0.48 | 0.39 | 0.17 |
| **Hip OA (K=0.03)** | |  |  |  |
|  | OR=1.05 | 0.08 | 0.06 | 0.06 |
|  | OR=1.10 | 0.17 | 0.11 | 0.10 |
|  | OR=1.20 | 0.50 | 0.30 | 0.26 |
|  | OR=1.30 | 0.83 | 0.57 | 0.51 |
|  | OR=1.40 | 0.97 | 0.82 | 0.75 |
| **Knee OA (K=0.05)** | |  |  |  |
|  | OR=1.05 | 0.10 | 0.07 | 0.05 |
|  | OR=1.10 | 0.24 | 0.15 | 0.14 |
|  | OR=1.20 | 0.70 | 0.45 | 0.39 |
|  | OR=1.30 | 0.96 | 0.78 | 0.72 |
|  | OR=1.40 | 1.00 | 0.95 | 0.92 |

All power calculations estimated based on an R^2^_xz_ (*i.e.* the proportion of variance in the exposure explained by the instrument) of 0.01 and a type-1 error rate of 0.05. K=proportion of cases. ^a^K=0.010 for females and 0.004 for males. OA: osteoarthritis; OR: odds ratio

## **Supplementary Table S4: Summary statistics for IGF-1 instruments used for two-sample MR analyses**

| **SNP** | **Annotated gene** | **EA** | **NEA** | **IGF-1** | | | | **Hip OA** | | | | **Knee OA** |  |  |
| --- | --- | --- | --- | --- | --- | --- | --- | --- | --- | --- | --- | --- | --- | --- |
|  |  |  |  | **EAF** | **Beta** | **SE** | ***P*** | **EAF** | **Beta** | **SE** | ***p*** | **Beta** | **SE** | ***p*** |
| rs1065656^*^ | *NUBP2* | C | G | 0.306 | -0.054 | 0.009 | 1.17x10^-8^ | 0.303 | 0.006 | 0.013 | 0.265 | -0.006 | 0.010 | 0.568 |
| rs2153960 | *FOXO3* | A | G | 0.687 | 0.055 | 0.009 | 5.16x10^-9^ | 0.711 | 0.027 | 0.013 | 0.033 | 0.023 | 0.010 | 0.028 |
| rs509035 | *GHSR* | A | G | 0.308 | 0.051 | 0.009 | 2.09x10^-8^ | 0.317 | 4x10^-4^ | 0.013 | 0.976 | 0.024 | 0.010 | 0.017 |
| rs646776^*^ | *CELSR2* | T | C | 0.785 | -0.028 | 0.010 | 6.87x10^-9 a^ | 0.778 | -0.040 | 0.014 | 0.004 | -0.003 | 0.011 | 0.794 |
| rs700753^*^ | *TNS3* | C | G | 0.347 | -0.092 | 0.009 | 1.60x10^-23^ | 0.347 | 0.004 | 0.012 | 0.753 | 0.010 | 0.010 | 0.292 |
| rs780093 | *GCKR* | T | C | 0.409 | -0.065 | 0.009 | 2.19x10^-13^ | 0.383 | -0.031 | 0.012 | 0.010 | -0.023 | 0.010 | 0.014 |
| rs934073 | *ASXL2* | C | G | 0.711 | -0.053 | 0.009 | 6.48x10^-9^ | 0.692 | -0.014 | 0.013 | 0.265 | 0.006 | 0.010 | 0.563 |
| rs978458 | *IGF1* | T | C | 0.265 | 0.057 | 0.010 | 1.56x10^-10^ | 0.261 | 0.038 | 0.013 | 0.005 | 0.013 | 0.011 | 0.209 |

Summary statistics for IGF-1 from the GIANT consortium and for hip and knee OA from the GWAS in UK Biobank and arcOGEN. IGF-1 GWAS in each of the 17 cohorts (listed in (6)) were adjusted for age, Principal Components (PCs), study centre, stratified by sex and accounted for relatedness and laboratory batch where applicable (6).

^*^SNPs also associated with IGF-BP3 in the CHARGE meta-analysis (6).

The hip and knee OA GWAS were adjusted for age, sex genotyping chip and PCs.

^a^*p* value from bivariate analysis. *P* value used to estimate beta= 0.09.

EA: effect allele; NEA: alternative allele; EAF: effect allele frequency; SE: standard error

## **Supplementary Table S5: Associations between the 63 BMI SNPs and BMI in UK Biobank**

| **SNP** | **EA** | **Beta** | **SE** | ***P*** |
| --- | --- | --- | --- | --- |
| rs1000940 | G | 0.068 | 0.013 | 5x10^-8^ |
| rs10132280 | A | -0.107 | 0.013 | 2x10^-17^ |
| rs1016287 | C | -0.100 | 0.013 | 3x10^-15^ |
| rs10182181 | G | 0.159 | 0.012 | <1x10^-300^ |
| rs10733682 | G | -0.061 | 0.012 | 2x10^-7^ |
| rs10938397 | G | 0.152 | 0.012 | <1x10^-300^ |
| rs10968576 | G | 0.115 | 0.012 | 7x10^-21^ |
| rs11030104 | G | -0.181 | 0.014 | 8x10^-37^ |
| rs11057405 | A | -0.127 | 0.019 | 1x10^-11^ |
| rs11165643 | T | 0.086 | 0.012 | 2x10^-13^ |
| rs1167827 | G | 0.099 | 0.012 | 2x10^-17^ |
| rs11727676 | C | -0.036 | 0.019 | 0.061 |
| rs12286929 | G | 0.085 | 0.012 | 2x10^-13^ |
| rs12401738 | A | 0.082 | 0.012 | 4x10^-12^ |
| rs12429545 | A | 0.122 | 0.017 | 2x10^-12^ |
| rs12940622 | A | -0.089 | 0.012 | 1x10^-14^ |
| rs13021737 | G | 0.257 | 0.015 | <1x10^-300^ |
| rs13078960 | G | 0.087 | 0.014 | 1x10^-9^ |
| rs13107325 | T | 0.231 | 0.022 | 6x10^-26^ |
| rs13191362 | G | -0.093 | 0.017 | 9x10^-8^ |
| rs1516725 | C | 0.159 | 0.017 | 2x10^-21^ |
| rs1528435 | T | 0.077 | 0.012 | 1x10^-10^ |
| rs1558902 | A | 0.354 | 0.012 | <1x10^-300^ |
| rs16851483 | T | 0.173 | 0.023 | 9x10^-14^ |
| rs16951275 | C | -0.134 | 0.014 | 2x10^-22^ |
| rs17001654 | G | 0.063 | 0.016 | 1x10^-4^ |
| rs17024393 | C | 0.347 | 0.036 | 1x10^-21^ |
| rs17094222 | C | 0.070 | 0.014 | 5x10^-7^ |
| rs17405819 | C | -0.094 | 0.013 | 6x10^-14^ |
| rs17724992 | G | -0.070 | 0.013 | 9x10^-8^ |
| rs1808579 | T | -0.104 | 0.012 | 2x10^-19^ |
| rs1928295 | C | -0.058 | 0.012 | 6x10^-7^ |
| rs2033529 | G | 0.094 | 0.013 | 2x10^-13^ |
| rs2033732 | C | 0.050 | 0.013 | 2x10^-4^ |
| rs205262 | G | 0.143 | 0.013 | 2x10^-28^ |
| rs2112347 | G | -0.141 | 0.012 | 8x10^-32^ |
| rs2121279 | T | 0.054 | 0.017 | 0.002 |
| rs2176598 | C | -0.083 | 0.013 | 4x10^-10^ |
| rs2207139 | G | 0.199 | 0.015 | 2x10^-38^ |
| rs2245368 | T | -0.115 | 0.015 | 7x10^-14^ |
| rs2287019 | T | -0.154 | 0.015 | 7x10^-25^ |
| rs2365389 | T | -0.063 | 0.012 | 9x10^-8^ |
| rs2820292 | C | 0.090 | 0.012 | 9x10^-15^ |
| rs29941 | G | 0.072 | 0.012 | 4x10^-9^ |
| rs3101336 | C | 0.107 | 0.012 | 1x10^-19^ |
| rs3736485 | G | -0.076 | 0.012 | 6x10^-11^ |
| rs3817334 | T | 0.114 | 0.012 | 2x10^-22^ |
| rs3849570 | A | 0.053 | 0.012 | 1x10^-5^ |
| rs3888190 | A | 0.124 | 0.012 | 3x10^-26^ |
| rs4256980 | G | 0.080 | 0.012 | 3x10^-11^ |
| rs4740619 | C | -0.087 | 0.012 | 7x10^-14^ |
| rs543874 | G | 0.224 | 0.014 | <1x10^-300^ |
| rs6477694 | T | -0.052 | 0.012 | 2x10^-5^ |
| rs6567160 | C | 0.259 | 0.014 | <1x10^-300^ |
| rs657452 | G | -0.069 | 0.012 | 4x10^-9^ |
| rs6804842 | G | 0.059 | 0.012 | 5x10^-7^ |
| rs7138803 | A | 0.127 | 0.012 | 2x10^-26^ |
| rs7141420 | T | 0.096 | 0.012 | 8x10^-17^ |
| rs758747 | T | 0.052 | 0.013 | 6x10^-5^ |
| rs7599312 | A | -0.078 | 0.013 | 3x10^-9^ |
| rs7899106 | G | 0.122 | 0.026 | 4x10^-6^ |
| rs7903146 | T | -0.084 | 0.013 | 3x10^-11^ |
| rs9400239 | C | 0.076 | 0.013 | 2x10^-9^ |

Betas are the per-allele unit increase in BMI (kg/m^2^)

Adjusted for sex, genotyping chip and 10 PCs

SNP: single nucleotide polymorphism; EA: effect allele; SE: standard error

## **Supplementary Table S6: Associations between 292 height SNPs used in genetic risk score and height in UK Biobank**

*Provided in separate excel file*

Betas are the per-allele unit increase in height (cm)

Adjusted for sex, genotyping chip and 10 PCs

SNP: single nucleotide polymorphism; SE: standard error

|  | **1S MR** | | | **2S MR (IVW)** | | | **2S MR (MR-Egger)** | | | **MV MR^a^** | | |
| --- | --- | --- | --- | --- | --- | --- | --- | --- | --- | --- | --- | --- |
|  | **OR** | **95% CI** | ***P*** | **OR** | **95% CI** | ***p*** | **OR** | **95% CO** | ***p*** | **OR** | **95% CI** | ***p*** |
| Hand OA | 0.99 | 0.57, 1.70 | 0.958 | 0.90 | 0.57, 1.43 | 0.665 | 0.02 | 1.08x10^-4^, 2.36 | 0.203 | 1.00 | 0.58, 1.71 | 0.986 |
| Hip OA | 1.57 | 1.21, 2.02 | 0.001 | 1.49 | 1.21, 1.83 | 1.39x10^-4^ | 5.88 | 0.70, 49.13 | 0.200 | 1.49 | 1.16, 1.93 | 0.002 |
| Knee OA | 1.30 | 1.07, 1.58 | 0.008 | 1.33 | 1.10, 1.60 | 0.003 | 2.09 | 0.21, 21.04 | 0.576 | 1.22 | 1.00, 1.48 | 0.049 |

## **Supplementary Table S7: Results of MR analyses excluding the three SNPs robustly associated with IGF-BP3**

One-sample and multivariable MR using genetic risk scores. F-statistics for 1SMR and S-W conditional F statistics for MVMR>1500.

^a^adjusting for BMI

1S: one-sample; 2S: two-sample; MV: multivariable; MR: Mendelian randomization

## **Supplementary Table S8: association between IGF-1 genetic risk score and potential confounders of the IGF-1-OA relationship**

| **Covariate** | **Beta** | **95% CI** | ***p*** |
| --- | --- | --- | --- |
| Age (years) | -1.08x10^-4^ | -0.001, 0.001 | 0.787 |
| Male | -0.004 | -0.017, 0.009 | 0.537 |
| BMI (kg/m^2^) | 0.017 | 0.008, 0.025 | 1.34x10^-4^ |
| ERT (ever used) | 0.008 | 0.003, 0.014 | 0.002 |
| Age at menopause | -0.002 | -0.004, 3.89x10^-4^ | 0.104 |

Beta represents the unit increase in GRS per one-year increase in age and age at menopause or the difference between genders. For BMI, beta represents the unit increase in BMI per unit increase in GRS. For ERT, beta represents the log odds of ever taking ERT per unit increase in GRS. Adjusted for sex, genotyping chip and 10 PCs. CI: confidence interval; BMI: body mass index, ERT: estrogen replacement therapy

|  |  | **IVW** | | | **Weighted median** | | | **MR-Egger** | | |
| --- | --- | --- | --- | --- | --- | --- | --- | --- | --- | --- |
|  |  | **OR** | **95% CI** | ***p*** | **OR** | **95% CI** | ***p*** | **OR** | **95% CI** | ***p*** |
| All SNPs (n_SNPs_=5) | Hand OA | 0.88 | 0.75, 1.02 | 0.090 | 0.82 | 0.69, 0.97 | 0.021 | 0.72 | 0.52, 0.99 | 0.136 |
|  | Hip OA | 1.00 | 0.90, 1.10 | 0.965 | 1.01 | 0.94, 1.08 | 0.808 | 1.15 | 0.99, 1.33 | 0.156 |
|  | Knee OA | 0.97 | 0.92, 1.02 | 0.284 | 0.97 | 0.92, 1.03 | 0.297 | 0.98 | 0.88, 1.10 | 0.799 |
| Excluding IGF-1 SNPs (n_SNPs_=2) | Hand OA | 0.85 | 0.64, 1.13 | 0.272 |  |  |  |  |  |  |
|  | Hip OA | 1.03 | 0.95, 1.11 | 0.491 |  |  |  |  |  |  |
|  | Knee OA | 0.97 | 0.91, 1.03 | 0.375 |  |  |  |  |  |  |

## **Supplementary Table S9: Two-sample Mendelian randomization results for the causal effect of IGF-BP3 on hospital-diagnosed OA outcomes**

IGF-BP3 SNPs: rs11977526, rs700753, rs1065656, rs4234798, rs646776 (6)

IVW: inverse variance weighted; SNPs: single nucleotide polymorphisms; OA: osteoarthritis

## **Supplementary figure legend**

## **Supplementary Figure S1: flowchart detailing sample size derivation for the observational and mendelian randomization populations**

## **Supplementary Figure S2: observational and one-sample MR results for hip OA, stratified by sex** Points represent the odds ratio per SD increase in IGF-1 concentration. Horizontal bars represent 95% confidence intervals. Observational analyses adjusted for age, sex, ERT, ethnicity. MR analyses adjusted for sex, genotyping chip and 10 principal components.

BMI: body mass index; 1SMR: one-sample mendelian randomization; MVMR: multivariable mendelian randomization

## **Supplementary Figure S3: Plots comparing IVW, weighted median and MR-Egger estimates for the association between IGF-1 and OA** Points represent the odds ratio per SD increase in IGF-1 concentration. Horizontal bars represent 95% confidence intervals.

## IVW: inverse-variance weighted

## **Supplementary Figure S4: Comparison of IVW, weighted median and MR-Egger estimates for IGF-1 and hip OA**

MR: Mendelian randomization; SNP: single nucleotide polymorphism; OA: osteoarthritis

## **Supplementary Figure S5: Gender-stratified factorial MR analyses of the interaction between IGF-1 and BMI on OA risk**

GRS: genetic risk score; BMI: body mass index; CI: confidence interval

# Supplementary References

1. Bycroft C, Freeman C, Petkova D, Band G, Elliott LT, Sharp K, et al. The UK Biobank resource with deep phenotyping and genomic data. Nature. 2018;562(7726):203-9.

2. Huang J, Howie B, McCarthy S, Memari Y, Walter K, Min JL, et al. Improved imputation of low-frequency and rare variants using the UK10K haplotype reference panel. Nature communications. 2015;6:8111.

3. Howie B, Marchini J, Stephens M. Genotype imputation with thousands of genomes. G3 (Bethesda). 2011;1(6):457-70.

4. Manichaikul A, Mychaleckyj JC, Rich SS, Daly K, Sale M, Chen WM. Robust relationship inference in genome-wide association studies. Bioinformatics. 2010;26(22):2867-73.

5. Wood AR, Esko T, Yang J, Vedantam S, Pers TH, Gustafsson S, et al. Defining the role of common variation in the genomic and biological architecture of adult human height. Nature genetics. 2014;46(11):1173-86.

6. Teumer A, Qi Q, Nethander M, Aschard H, Bandinelli S, Beekman M, et al. Genomewide meta-analysis identifies loci associated with IGF-I and IGFBP-3 levels with impact on age-related traits. Aging cell. 2016;15(5):811-24.

# Supplementary References

1. Bycroft C, Freeman C, Petkova D, Band G, Elliott LT, Sharp K, et al. The UK Biobank resource with deep phenotyping and genomic data. Nature. 2018;562(7726):203-9.

2. Huang J, Howie B, McCarthy S, Memari Y, Walter K, Min JL, et al. Improved imputation of low-frequency and rare variants using the UK10K haplotype reference panel. Nature communications. 2015;6:8111.

3. Howie B, Marchini J, Stephens M. Genotype imputation with thousands of genomes. G3 (Bethesda). 2011;1(6):457-70.

4. Manichaikul A, Mychaleckyj JC, Rich SS, Daly K, Sale M, Chen WM. Robust relationship inference in genome-wide association studies. Bioinformatics. 2010;26(22):2867-73.

5. Wood AR, Esko T, Yang J, Vedantam S, Pers TH, Gustafsson S, et al. Defining the role of common variation in the genomic and biological architecture of adult human height. Nature genetics. 2014;46(11):1173-86.

6. Teumer A, Qi Q, Nethander M, Aschard H, Bandinelli S, Beekman M, et al. Genomewide meta-analysis identifies loci associated with IGF-I and IGFBP-3 levels with impact on age-related traits. Aging cell. 2016;15(5):811-24.
