# Supplementary figures and images for: Mendelian randomization provides evidence for a causal effect of higher serum IGF-1 concentration on risk of hip and knee osteoarthritis
Source: Rheumatology (Oxford). 2020 Oct 7;60(4):1676–86. doi: 10.1093/rheumatology/keaa597 (PMC8023994; doi:10.1093/rheumatology/keaa597)

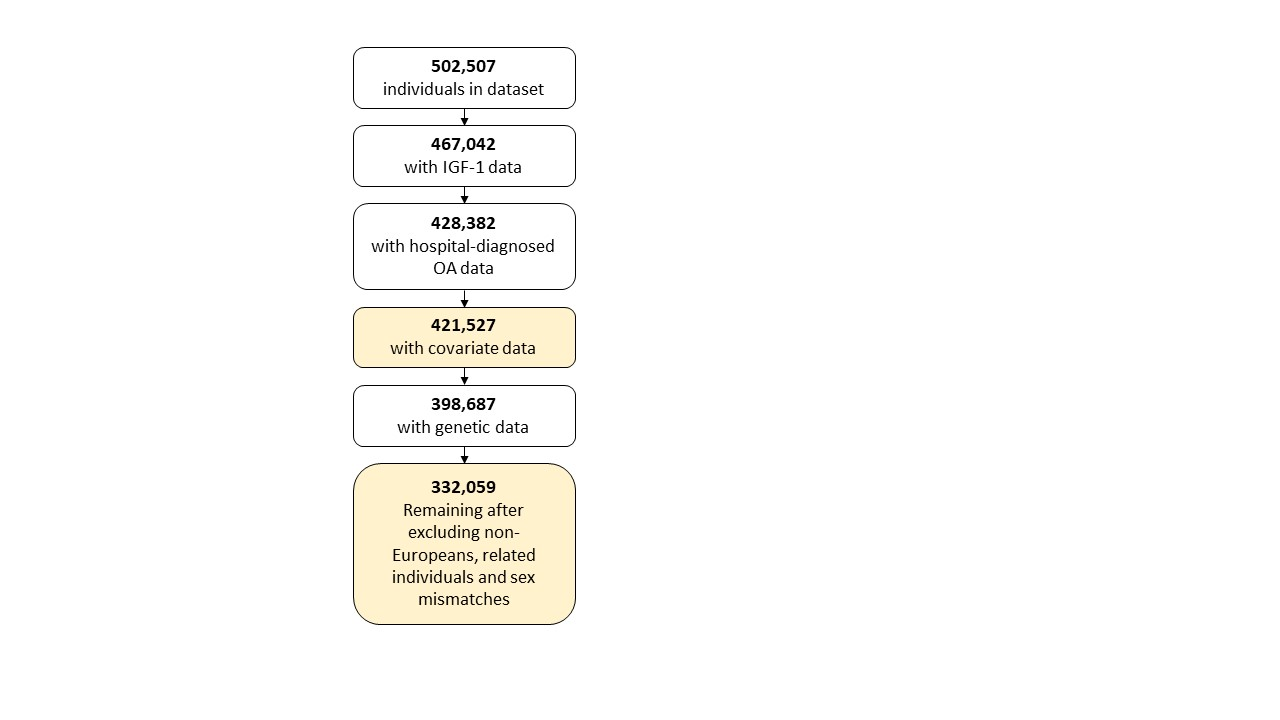

Supplement: keaa597_Supplementary_Data [file keaa597_supplementary_data.zip › keaa597-suppl_data/rhe-20-0365-File007.tif]

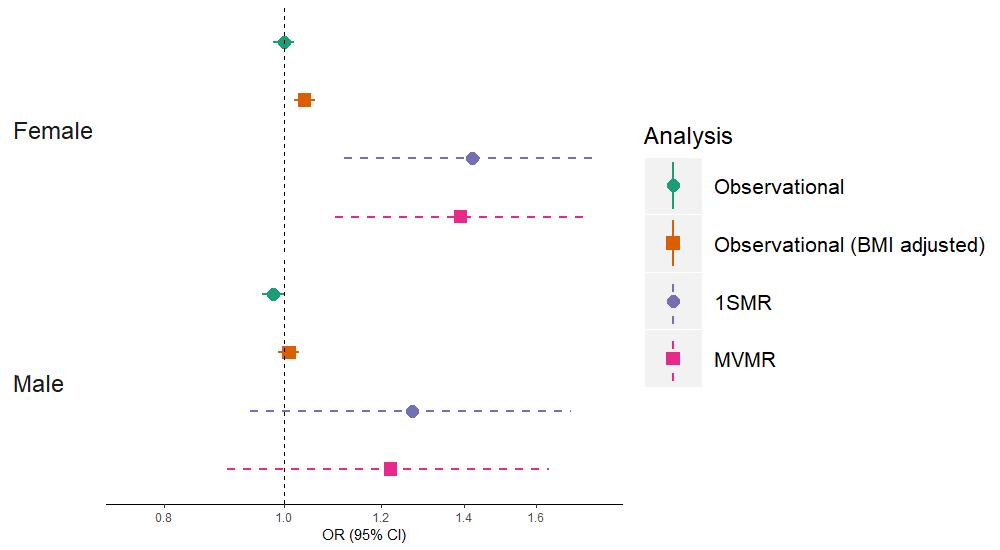

Supplement: keaa597_Supplementary_Data [file keaa597_supplementary_data.zip › keaa597-suppl_data/rhe-20-0365-File008.tif]

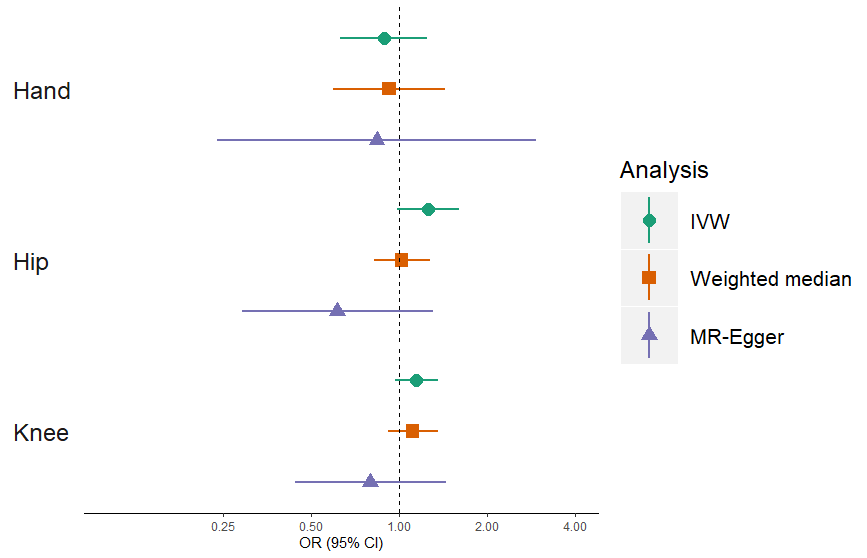

Supplement: keaa597_Supplementary_Data [file keaa597_supplementary_data.zip › keaa597-suppl_data/rhe-20-0365-File009.tif]

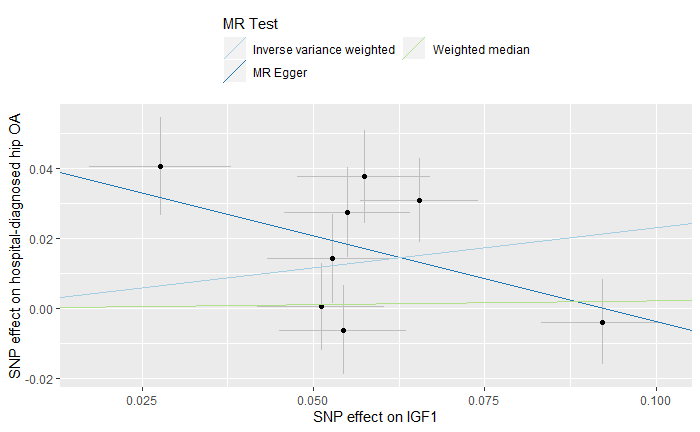

Supplement: keaa597_Supplementary_Data [file keaa597_supplementary_data.zip › keaa597-suppl_data/rhe-20-0365-File010.tif]

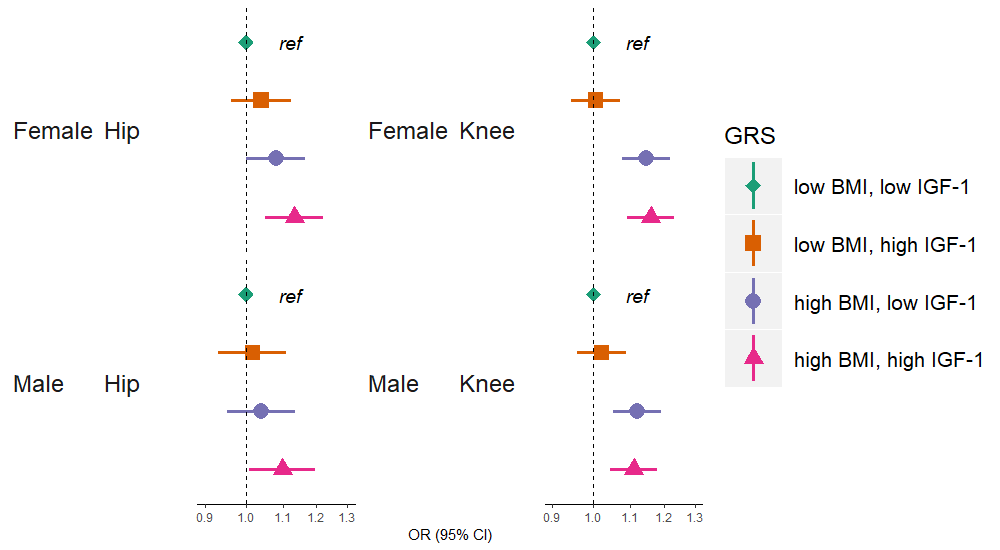

Supplement: keaa597_Supplementary_Data [file keaa597_supplementary_data.zip › keaa597-suppl_data/rhe-20-0365-File011.tif]
